# The specificity landscape of bacterial ribonuclease P

**DOI:** 10.1016/j.jbc.2023.105498

**Published:** 2023-11-25

**Authors:** Alexandra R. Chamberlain, Loc Huynh, Wei Huang, Derek J. Taylor, Michael E. Harris

**Affiliations:** 1 Department of Chemistry, University of Florida, Gainesville, Florida, USA; 2 Department of Pharmacology, Case Western Reserve University School of Medicine, Cleveland, Ohio, USA

**Keywords:** RNA processing, mutagenesis, endonuclease, enzyme kinetics, nucleic acid enzymology, ribonuclease, RNA

## Abstract

Developing quantitative models of substrate specificity for RNA processing enzymes is a key step toward understanding their biology and guiding applications in biotechnology and biomedicine. Optimally, models to predict relative rate constants for alternative substrates should integrate an understanding of structures of the enzyme bound to “fast” and “slow” substrates, large datasets of rate constants for alternative substrates, and transcriptomic data identifying *in vivo* processing sites. Such data are either available or emerging for bacterial ribonucleoprotein RNase P a widespread and essential tRNA 5′ processing endonuclease, thus making it a valuable model system for investigating principles of biological specificity. Indeed, the well-established structure and kinetics of bacterial RNase P enabled the development of high throughput measurements of rate constants for tRNA variants and provided the necessary framework for quantitative specificity modeling. Several studies document the importance of conformational changes in the precursor tRNA substrate as well as the RNA and protein subunits of bacterial RNase P during binding, although the functional roles and dynamics are still being resolved. Recently, results from cryo-EM studies of *E. coli* RNase P with alternative precursor tRNAs are revealing prospective mechanistic relationships between conformational changes and substrate specificity. Yet, extensive uncharted territory remains, including leveraging these advances for drug discovery, achieving a complete accounting of RNase P substrates, and understanding how the cellular context contributes to RNA processing specificity *in vivo*.

RNA processing enzymes, including ribonuclease P (RNase P), are mediators of RNA metabolism and often participate in multiple pathways. The substrate specificities of RNA processing RNases like P, III, E, and others have been well studied (1, 2, 3, 4, 5). Yet, we are just beginning to appreciate mechanistically how they distinguish between multiple alternative substrates within the transcriptome. High-throughput and transcriptome-wide methods combined with comprehensive modeling have dramatically advanced our understanding of the biological roles and specificity of RNA-binding proteins (RBPs) (6, 7). Developing and applying quantitative models delineating RNA recognition have the potential to reveal fundamental RBP specificity rules and identify new functional characteristics (8, 9, 10). RNases and RNA processing enzymes contain and, in some cases, define classes of widespread and essential RBPs (11, 12, 13). However, because they undergo catalytic turnover, a significant challenge is integrating the kinetics of RNA processing enzymes into comprehensive models of RNA specificity that have similar potential to expand our understanding of biology and biomedicine.

The Specificity Constant (*k*_cat_/*K*_m_) which expresses the rate at which an enzyme combines with a substrate to form a product is the basis for quantifying relative rates of processing of alternative substrates (14). Differences in *k*_cat_/*K*_m_ largely reflect the degree of conformity to a set of optimal substrate RNA sequences and structures (*i.e.*, inherent specificity). However, intrinsic specificity can be modulated by multiple factors in the cell, resulting in the biological specificity that operates *in vivo* (Fig. 1) (15, 16). These factors include local RNA structure and concentration, competition with other processing enzymes or RBPs, and cellular localization (*i.e.*, compartmentalization) (2, 12). RNase P is a Mg^2+^ ion-dependent endonuclease that processes the 5′ ends of all tRNA precursors (pre-tRNAs) and other non-tRNA substrates in the transcriptome (3, 17). Thus, RNase P discriminates between cognate (*i.e.*, biologically relevant substrates) and non-cognate substrates but also accommodates variation among cognate substrates (18). Because of such broad specificity, understanding the mechanisms of RNA processing enzymes requires quantitative models of specificity that can accommodate a continuum of *k*_cat_/*K*_m_ values and account for the multiple factors that contribute to biological specificity (15, 19). Thus, comprehensive datasets of *k*_cat_/*K*_m_ values for pools of alternative substrates and transcriptome-wide data aimed at identifying *in vivo* substrates provide foundational information for developing quantitative models of global RBP specificity (19). While continually expanding, these datasets have begun to emerge for bacterial RNase P, making it a valuable model for exploring how intrinsic and biological specificity dictate the biological roles of RNA processing enzymes.

**Figure 1:**
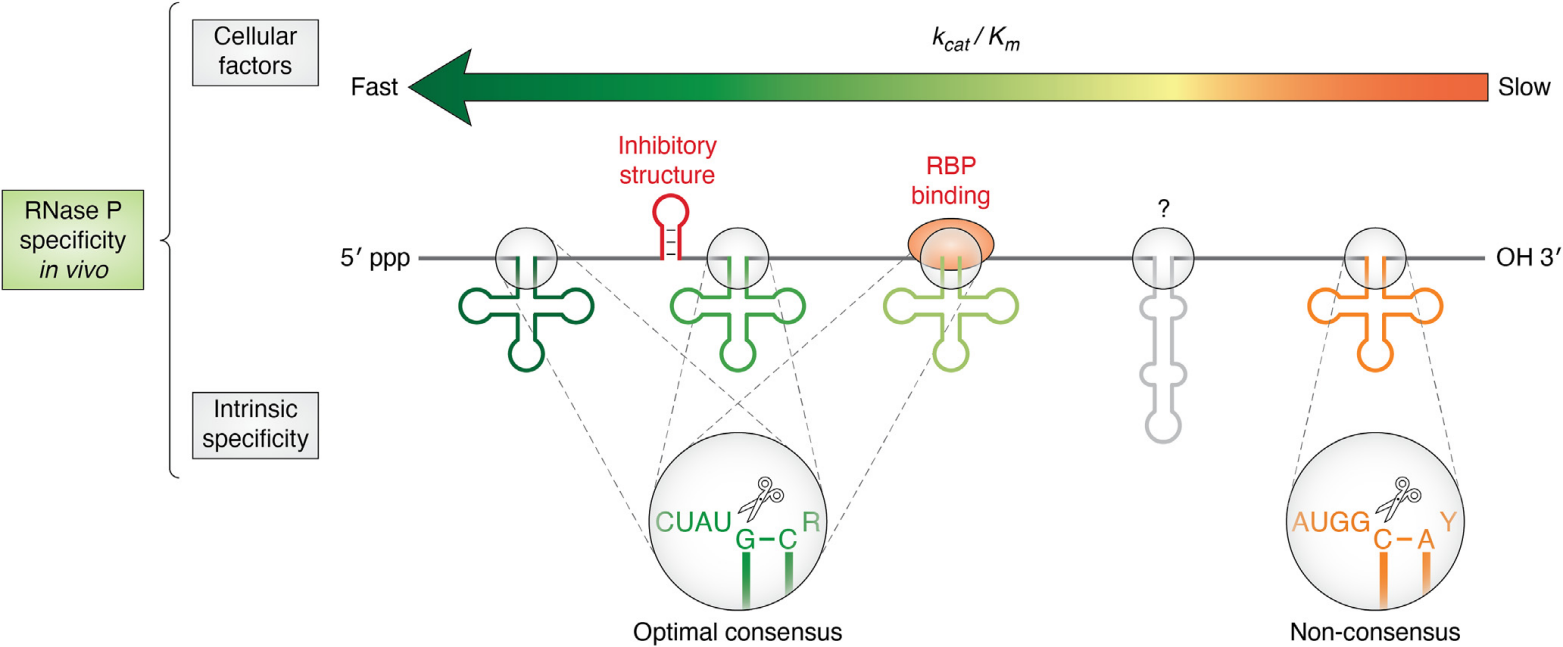
**Complex specificity landscape for RNase P substrate recognition.** RNA processing enzymes like RNase P are driven by “intrinsic specificity” determined by consensus sequences that are optimal and non-consensus sequences that lack a complete set of optimal binding interactions. In addition, the flanking sequence context can alter relative reaction rates through inhibitory structure, RBP binding, or other “cellular factors” that together with intrinsic specificity give rise to the observed RNase P specificity *in vivo*. Optimally, predictive models of specificity can accommodate both contributions, relate them to relative processing rates, and identify novel substrates (*gray*, question mark).

## Understanding the ability to recognize and bind to multiple substrates is critical to defining RNase P specificity

RNase P is fundamentally a multiple-substrate enzyme and is broadly representative of RNA processing enzymes that act on many substrates in the transcriptome. Bacterial RNase P recognizes both monocistronic and polycistronic RNA precursors as substrates (20, 21, 22, 23). In *Escherichia* coli, 26 of the 86 tRNAs are either monocistronic precursors or occur as the first tRNA in a polycistronic transcript with 5′ leader sequences varying from 2 to 52 nucleotides (24, 25). tRNA genes are more extensively clustered in some Gram-positive bacteria (26, 27). For example, the 69 tRNA genes in *Bacillus subtilis* are grouped into only nine polycistronic units (28). The extent to which RNase P is engaged in separating different bacterial polycistronic pre-tRNAs is not known. Another key determining factor for RNase P recognition (as described below) involves the 3′ RCCA sequence of pre-tRNA, even though it is not encoded in approximately one-third of *B. subtilis* tRNA precursors (29). This pattern is similarly observed in *Staphylococcus aureus* tRNA precursors, where 17 of 60 tRNA precursors lack the 3′ CCA sequence.

Multiple factors including processing are involved in setting appropriate steady state levels of tRNAs and responding to environmental changes by regulating tRNA pools. While a full discussion is beyond the scope, it is important to note that gene dosage (30) and chromosomal position effects (31) influence the expression of tRNAs. Expression is regulated by the stringent response leading to the downregulation of stable RNA synthesis including tRNAs (32). The modification of tRNAs is both extensive and highly dynamic in response to environmental conditions (33, 34). Concerning the biological specificity of RNase P and regulation, microarray studies revealed that steady-state tRNA levels are determined by transcription, processing efficiency, and turnover of tRNA precursors (35). In contrast, there is little evidence that regulation involving the degradation of mature tRNAs plays a significant role.

**Figure 2:**
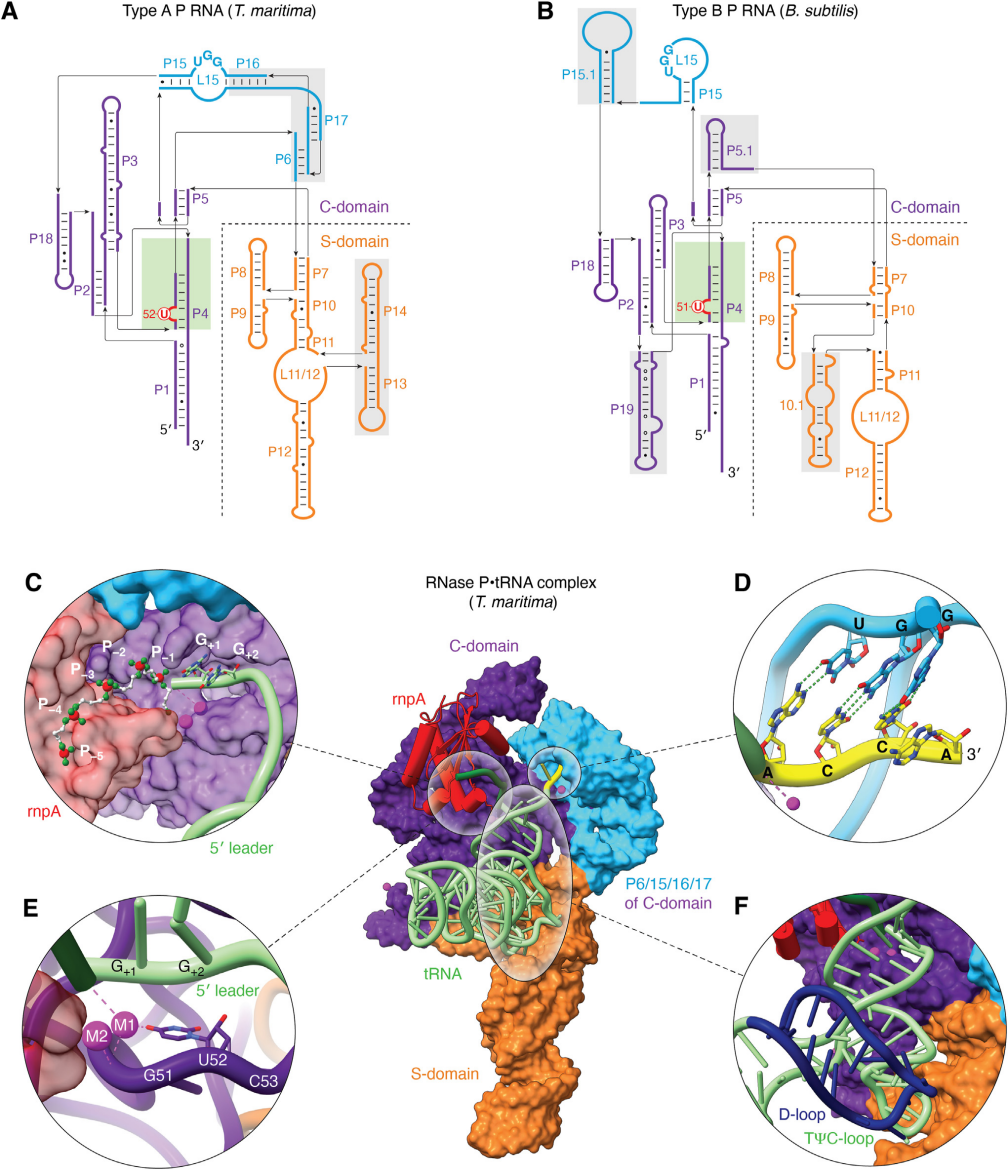
**Recognition of intrinsic specificity elements of a pre-tRNA by bacterial RNase P.***Top panel*: Secondary structures of bacterial RNase P RNAs. *A*, Type A – *T. maritima*. *B*, Type B – *B. subtilis*. Primary differences are highlighted in *gray boxes*. The most conserved region P4 helix (highlighted in *green*) houses the universally conserved bulged U (*red*). *Bottom panel*: The crystal structure of the *Thermotoga maritima* RNase P•tRNA complex and 5′ leader. S-domain: *orange*, C-domain: *purple*, and P6/15/16/17 regions: *cyan*. *C*, close-up view of binding of 5′ leader phosphate backbone (phosphorous: *red*, oxygen: *green*, carbon: *white*) of tRNA at the protein (rnpA) interface and S-domain of P RNA subunits. *D*, close-up view of tRNA recognition by RNase P by Watson-Crick base pairs between GGU of L15 loop and ACC of 3′ tRNA. *E*, close-up view at the active site where the universally conserved bulged U52 nucleotide in the P4 helix (C-domain) binds catalytic metal ions (*magenta*). *F*, close-up view of RNase P recognizing tRNA by the length of tRNA acceptor stem and T-stem. P9/10/11 of S-domain contact TΨC-loop of tRNA.

The structure determinants recognized by RNase P enzymes across phylogeny appear highly similar, as expertly covered in the review by Lei and colleagues (in this issue). RNase P from representative archaea and eukaryotes are well understood at a structural level, and extensive experimental studies of bacterial RNase P specificity, including quantitative high-throughput analyses, provide a necessary framework for the development of predictive models of specificity (reviewed in (3, 17, 36, 37, 38)). Importantly, bacterial RNase P occurs in two distinct secondary structure classes; the first is Type A, referred to as the “ancestral” class while Type B evolved later among Bacteria (39). The crystal structure of the *Thermotoga maritima* RNase P•tRNA complex (40) and the recent cryo-EM structure of the *E. coli* RNase P•pre-tRNA complex (41) paint a consistent picture of the interactions that contribute to the intrinsic specificity of bacterial Type A RNase P (Fig. 2*A*). Type B, typified by *B. subtilis* RNase P (Fig. 2*B*), retains the conserved core found in all P RNA subunits but differs in structures that contribute to stability as well as substrate recognition (39) the functional implications of which Are not fully understood.

Extensive structure-function studies of bacterial RNase P demonstrate that both Type A and B recognize optimal sequences flanking the cleavage site and the 3′ RCCA of tRNA, which helps to measure the appropriate length of the acceptor stem for proper processing (Fig. 2, *C*–*F*). Within the S-domain of the P RNA subunit, P9, P10, and P11 contact the TΨC-loop of tRNA. Interactions near the cleavage site involve sequence-specific contacts to the 5′ leader and 3′RCCA sequence. The 3′RCC of the RCCA motif of tRNA forms Watson-Crick base pairs with a GGU sequence in the P15 internal loop (L15) of RNase P. Both *E. coli* and *B. subtilis* RNase Ps cleave the pre-tRNA between N(+1) and N(-1). Similarly, enzymes of both species prefer uracil at N(-1) in the 5′ leader of pre-tRNA due to steric constraints and H-bonding formed between the uracil and A248 (in *E. coli*) in J5/15 of P RNA (42, 43, 44). The P protein subunit, which will be referred to by its gene name rnpA, interacts with leader nucleotides distal to N(-3) and overall increases the affinity for metal ions essential for catalysis, which in turn may act to suppress the effects of variation in intrinsic specificity determinants (45, 46). Additionally, the N(-4) nucleobase is recognized by conserved aromatic residues in rnpA, resulting in hydrophobic interactions providing enhanced affinity with minimal sequence specificity (41). The P4 helix in the C-domain is the most conserved region of P RNA and positions a universally conserved bulged U that, together with residues in J3/4, coordinates catalytic metal ions (47, 48, 49).

Other factors that affect *k*_cat_/*K*_m_ *in vitro* involve identifying flanking 5′ and 3′ sequences. However, their contributions to biological specificity are only understood in a few cases. Multiple lines of investigation show that pairing of proximal 5′ leader nucleotides with the 3′ RCCA of pre-tRNA acts as an anti-determinant for RNase P cleavage (50, 51, 52, 53, 54, 55). In fact, a significant number of pre-tRNAs in the genome of *E. coli* have G(-2)G(-1) or G(-2)U(-1) (4 or 18, respectively), which results in extension of the acceptor stem that can slow *k*_cat_/*K*_m_ (41). Importantly, a mismatch at position N(-1) is a key determinant for pre-tRNA processing by eukaryotic nuclear RNase P, presumably because it blocks the formation of an extended acceptor stem (56). The length and presence of stable secondary structure in the 5′ leader proximal to the RNase P cleavage site also affects *k*_cat_/*K*_m_ (24, 50). The effect of multiple adjacent pre-tRNAs in polycistronic precursors, which are separated by as few as two nucleotides, has not been systematically examined. However, the 3′ to 5′ directionality of RNase P processing of polycistronic pre-tRNA observed *in vivo* is reproduced *in vitro* for model polycistronic pre-tRNA (25, 57). Thus, differences in processing rates of different pre-tRNA substrates can in part be attributed to intrinsic specificity, as opposed to factors that operate only in the cell. However, in general variation at the nucleotides flanking the cleavage site and the 5′ leader sequence has only a few-fold effect on *k*_cat_/*K*_m_ (58, 59). In contrast, contributions from pairing of the proximal 5′ leader with the 3′ RCCA or distal 5′ leader sequences tend to be more significant, often resulting in orders of magnitude differences in relative rate constants for RNase P cleavage *in vitro* (25, 50, 60).

**Figure 3:**
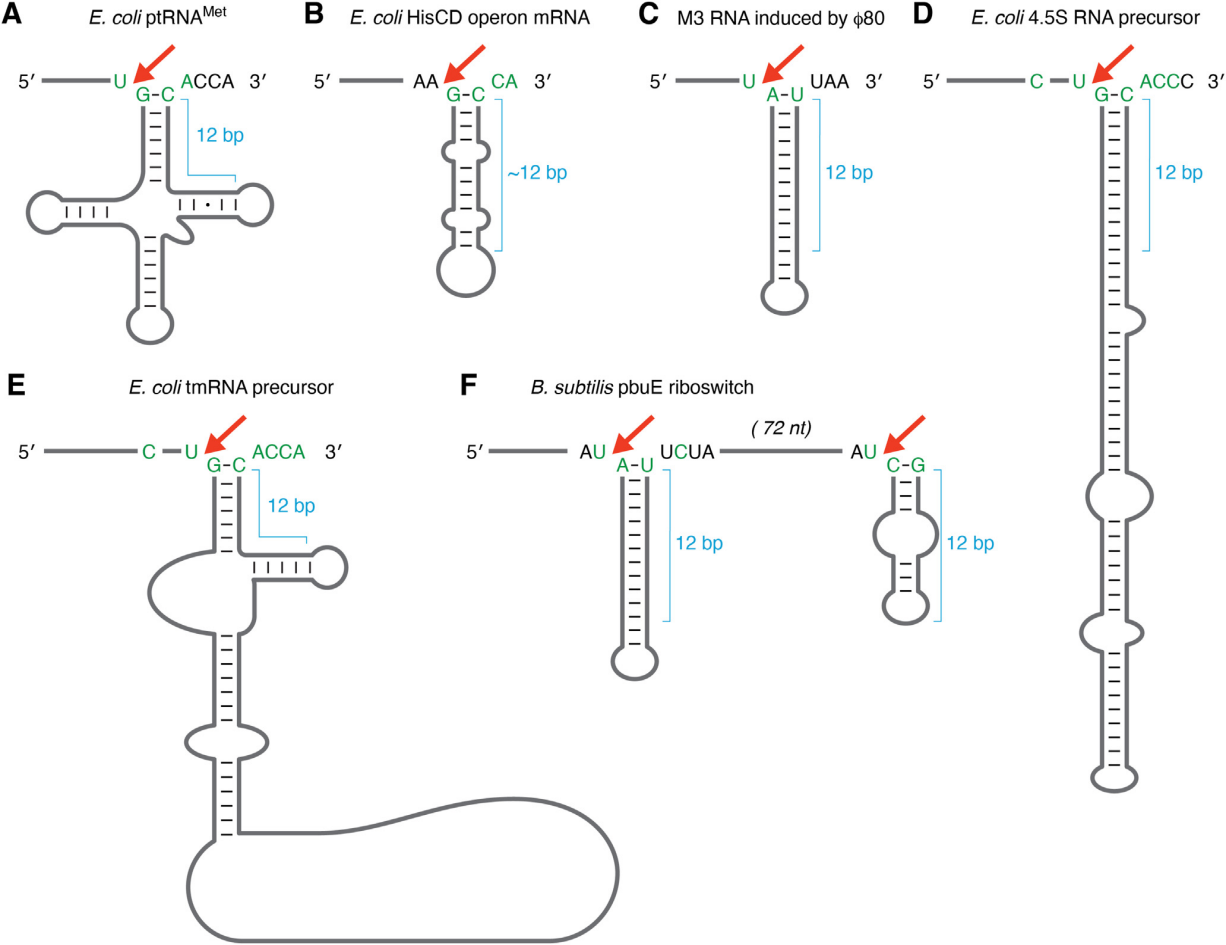
**Intrinsic pre-tRNA specificity determinants are present in non-pre-tRNA substrates of bacterial RNase P.** The pre-tRNA and non-pre-tRNA substrates of RNase P in *E. coli* are shown with optimal consensus recognition elements determined for pre-tRNA substrates *in vitro* are colored green. A bracket adjacent to the helical regions of substrates represents the 12 bp length measured by RNase P for pre-tRNA substrates. The proximal 5′ leader length of 10 nucleotides is shown for each substrate, and a red arrow indicates the RNase P cleavage site. *A*, *E. coli* pre-tRNA^met^. *B*, *E. coli* HisCD operon mRNA *C*, M3 RNA induced by bacteriophage phi80. *D*, *E. coli* 4.5 S RNA precursor. *E*, *E. coli* tmRNA precursor. *F*, *B. subtilis* pbuE riboswitch.

In addition to pre-tRNAs, bacterial RNase P also processes the precursors of the signal recognition particle RNA (SRP RNA) (61) and transfer-messenger RNA (tmRNA) (62, 63). Additionally, RNase P cleaves several mRNAs and small RNAs associated with phage and plasmid replication (Fig. 3) (64). As with *E. coli* RNase P, the cleavage site for pre-tmRNA matches the optimal consensus derived for pre-tRNA, including a 12-nucleotide stem, TΨC loop, and 3′ RCCA, all of which contribute to its function as a tRNA mimetic. In addition, an optimal U(-1)/G(1) sequence resides at the pre-tmRNA cleavage site with C(-4) in the 5′ leader. The pre-SRP RNA cleavage site matches the optimal consensus for *E. coli* RNase P pre-tRNA recognition; however, a continuous dsRNA helix replaces the TΨC loop. Similarly, the C4 bacteriophage RNA has an extended stem instead of a TΨC loop sequence and a cleavage site that matches the optimal consensus for RNase P, except for C(-4). Thus, interactions with J10/11 in the S-domain of RNase P do not exist for C4 bacteriophage RNA and pre-SRP RNA substrates, which would likely affect *k*_cat_/*K*_m_ depending on the precise step that is rate limiting. In the HisCD operon, *E. coli* RNase P cleavage of an RNA hairpin is proposed to stabilize a portion of the polycistronic mRNA (65). The HisCD cleavage site partially matches the consensus derived from pre-tRNA studies. The pbuE adenine riboswitch is a substrate for *B. subtilis* RNase P *in vitro* and depletion of RNase P results in reduced expression of a lacZ positioned downstream of pbuE (66). The current model for *E. coli* RNase P specificity predicts that it is unlikely that these alternative substrates could compete with more abundant pre-tRNA substrates. Despite having a solid understanding of RNase P processing of pre-tRNA substrates, the kinetics of processing alternative non-pre-tRNA substrates are much less clear.

In principle, a comprehensive model of RNase P specificity should enable the prediction of potential non-canonical substrates within the transcriptome. Transcriptome-wide analysis of RNase P substrates using microarrays to identify RNAs that accumulate in a yeast mutation strain defective in nuclear RNase P was an early milestone. These studies revealed roles in the turnover of an rRNA and cleavage of a subset of snoRNAs (67, 68, 69). RNase P-dependent changes in yeast RNA abundance were also examined by sequencing RNAs copurified with RNase P and probed directly by northern blot (70, 71). Recently, Kushner and colleagues studied transcriptome-wide changes in *E. coli* gene expression resulting from RNase P depletion using a temperature-sensitive *E. coli* mutant of the rnpA protein (72). In addition to the accumulation of known RNase P substrates, loss of RNase P activity also affected a significant fraction of mRNAs, suggesting a possible role in mRNA turnover. While direct connections to specific RNase P cleavage sites remain to be established, these results underscore the broad importance of RNase P beyond pre-tRNA processing.

Concerning biological specificity, several studies show that deletion of the endogenous bacterial RNase P protein subunit (rnpA) can be rescued by heterologous gene versions. Replacement of *E. coli* rnpA with other bacterial rnpA genes revealed that proteins with divergent sequences can rescue cell growth, albeit with significant defects in fitness (73, 74). *E. coli* cells expressing chimeric RNase P enzymes exhibit altered growth rates and changes in their transcriptomes that have yet to be fully explored (73, 75). Remarkably, nuclear and organellar protein-only RNase P (PRORP) from *Arabidopsis* can replace *E. coli* RNase P and maintain cell viability (76). However, RNA-Seq analyses show that *Arabidopsis* PRORP1 cleaves several pre-tRNAs aberrantly, primarily those with short acceptor stem extensions, and fails to process precursor SRP RNA which nonetheless functions in these forms. This finding suggests that these incorrectly processed and unprocessed RNAs do not alter functions essential to cell survival. Depletion of RNase P in *E. coli* leads to the accumulation of specific polycistronic pre-tRNAs as well as incorrectly processed tRNAs. The observation that aberrantly processed tRNA can still be aminoacylated suggests an essential function of RNase P in *E. coli* may be the separation of polycistronic pre-tRNAs (77), and there appears to be remarkable flexibility intrinsic to the enzyme for this function. Thus, the essential function of ribonucleoprotein RNase P is replaceable, but optimal fitness under diverse conditions is affected by the substrate specificity of the endogenous RNase P enzyme.

## Quantitative modeling of alternative substrate specificity

**Figure 4:**
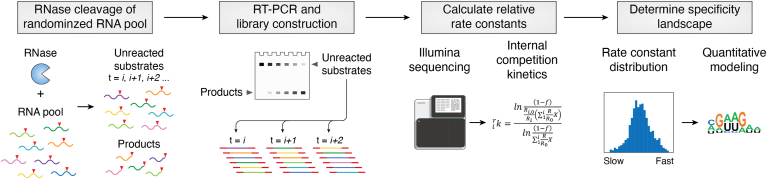
**High-throughput sequencing kinetics (HTS-Kin) analysis and quantitative modeling specificity.** HTS-Kin is one method for the determination of rate constant distributions of randomized populations useful for modeling substrate specificity. In this approach, an RNase like RNase P is reacted with a pool of randomized RNA substrate designed to interrogate a specific region or set of interactions. The unreacted substrates from different time points are isolated by gel purification and RT-PCR is used to construct libraries for Illumina sequencing. The relative rate constants for all members of the population are determined from the change in a number of sequence reads using internal competition kinetics. The resulting distribution of rate constants serves as the basis for the development of comprehensive models of substrate sequence and structure specificity.

A powerful approach to define enzyme specificity is to experimentally determine relative processing rates *in vitro* for many alternative substrates. Unique challenges for applying this approach include creating randomized substrate pools, quantifying changes in substrate and/or product populations, and extracting relative rate constants. To overcome these challenges for RNase P, we developed High Throughput Sequencing-Kinetics (HTS-Kin) to obtain large distributions of kinetic measurements for alternative substrates (Fig. 4*A*). These data allowed elements of inherent and biological specificity to be defined for *E. coli* RNase P against thousands of unique sequences (60, 78). Specifically, HTS-Kin measures the relative rate constants of randomized populations of RNA substrates in a single reaction using standard molecular biology methods and Illumina sequencing protocols (60, 79). Briefly, a population of RNA substrates containing a randomized sequence region is reacted with an RNA processing enzyme. The cleavage of the fastest reacting sequences results in their depletion from the substrate population early in the reaction. RNA sequences that react with lower *k*_cat_/*K*_m_ values are depleted more slowly. The relative *k*_cat_/*K*_m_ values for essentially all sequences in the population are calculated by quantifying the change in the number of sequence reads for each variant as a function of reaction progress. This technique's underlying principle uses internal competition kinetics to extract relative rate constants for many competing substrates in a combinatorial library tested in a single reaction mixture (60, 80, 81).

The initial application of HTS-Kin analyzed the specificity of *E. coli* rnpA with its corresponding binding site in the 5′ leader of pre-tRNA. The N(-2) to N(-8) positions of a canonical pre-tRNA^met^ were randomized, and the distribution of rate constants was determined for the population. Both biased and unbiased approaches help analyze such data sets to extract intrinsic specificity rules and deconvolute structural anti-determinants. The collection of substrates with the fastest rate constants can be used to define an optimal sequence logo, which identifies positive determinants but does not constitute a comprehensive specificity model. Simple sequence specificity models that use the entire data set based on position-weight matrices (PWM) provide a more comprehensive view (Fig. 4*B*). However, PWM modeling that treats each position in the binding site as independent and non-interacting fails to adequately describe the rate constant distributions determined by HTS-Kin. Including cooperativity factors (IC values) that express thermodynamic coupling terms between substrate nucleotides reflecting pairing or other interactions, however, provides more accurate unbiased models of RNA specificity (Fig. 4*C*). Applying this approach, we showed that unfavorable RNA structure involving the 5′ leader, revealed by significant IC values required for fitting, can significantly contribute to *k*_cat_/*K*_m_.

A primary function of rnpA is to position the 5′ leader of the pre-tRNA for processing. To fulfill this role the protein subunit interacts directly with more distal nucleotides of the leader, whereas P RNA positions the proximal nucleotide N(-1) and N(-2). To determine whether distal leader interactions with rnpA can modulate or alter RNA-RNA interactions with N(-1)N(-2), we compared the relative *k*_cat_/*K*_m_ values for pre-tRNA randomized from N(-1) to N(-6) for processing by the *E. coli* P RNA subunit alone and by the RNase P holoenzyme. While the P RNA subunit shows specificity for 5′ leader nucleotides N(−2) and N(−1), the presence of *E. coli* rnpA, reduces the contribution of P RNA to specificity, and alters specificity at N(−2) and N(−3) (52). The analysis also clearly showed that pairing the 5′ leader with the 3′ ACCA of tRNA acts as an anti-determinant for RNase P cleavage.

In addition to its well-characterized role in the 5′ end maturation of tRNAs, RNase P is required to separate pre-tRNAs from multiple polycistronic tRNA transcripts in *E. coli* (57, 77, 82). The 3′ to 5′ processing in polycistronic transcripts provides a valuable model system to dissect the effects of “biological specificity” focusing on the contribution of local RNA context. The simplest polycistronic pre-tRNA processed by *E. coli* RNase P is ValVW, a dicistronic transcript containing two copies of tRNA^Val^. This precursor is processed directionally *in vitro* allowing systematic structure-function studies to determine how 5′-leader and 3′-trailer sequences affect RNase P specificity. We demonstrated a distributive (*i.e*., non-processive) mechanism for directional processing of ValVW meaning RNase P dissociates after the first cleavage event and rebinds to catalyze the second. Additionally, we identified stem-loops flanking the 5′ proximal tRNA that inhibits cleavage and thereby enforces 3′ to 5′ directional processing. Structure-function studies confirmed that a stable stem-loop located two nucleotides from the beginning of the 5′ leader sequence in a monocistronic pre-tRNA results in a *ca*.10-fold reduction in the observed rate constant for RNase P processing (*e.g.*, see Fig. 1). Thus, the presence or absence of stable structure in the 5′ leader sequence within the P protein binding site and the specific sequence of the 5′ leader itself contribute to specificity.

## Understanding the mechanistic basis for specificity

The sequences and structures of alternative pre-tRNAs described above imply a limited degree of pre-organization in the free pre-tRNA relative to the bound state (18, 50, 83). Thus, conformational rearrangements, resulting in a catalytically active ES∗ complex, are required prior to or during association, introducing additional free energy barriers before forming an active ES∗ complex. Conformational changes can allow for the recognition and cleavage of a broader range of substrates or reduce the effects of variation depending on the details of the kinetic mechanism (84, 85). Considering how these principles apply to RNase P suggests that both mechanistic effects can contribute to specificity. This framework offers experimentally testable predictions and reveals additional challenges in quantitatively relating substrate structure to processing rate.

While the cryo-EM structures of human, yeast, and archaeal RNase P show that these enzymes are primarily pre-organized for efficient substrate binding (86, 87, 88), substrate binding by bacterial RNase P appears more dynamic. Early studies using minimal substrates showed that distal interaction between the D-/T-loop and the S-domain affects catalysis by *E. coli* RNase P, suggesting an induced-fit mechanism (89, 90). Inhibition of cleavage results from disrupting any combination of active-site contacts, metal-ion interactions, and 3′ RCCA pairing. The redundant interactions contribute to maintaining fidelity but could equally provide flexibility to recognize variations in sequence and structure inherent to multiple substrates *in vivo* (42). Stopped-flow studies of *B. subtilis* RNase P provided strong evidence for a two-step binding mechanism with an association step near the diffusion limit that forms a weak encounter complex (ES), followed by a conformational change linked to catalytic metal ion binding (ES∗) (91, 92). More recently, using pulse-chase experiments we provided evidence that pre-tRNA with an extended acceptor stem due to 3′ RCC pairing forms a greater fraction of ES versus ES∗. However, under conditions where catalysis is fast relative to the reverse rate constant for conversion of ES∗ back to ES, the effect on *k*_cat_/*K*_m_ is relatively small (41). Because pre-tRNAs with extended acceptor steps are frequent in the *E. coli* transcriptome, understanding the structural basis for the conformational changes in bacterial RNase P and pre-tRNA that occur during the formation of ES∗ is critically important.

**Figure 5:**
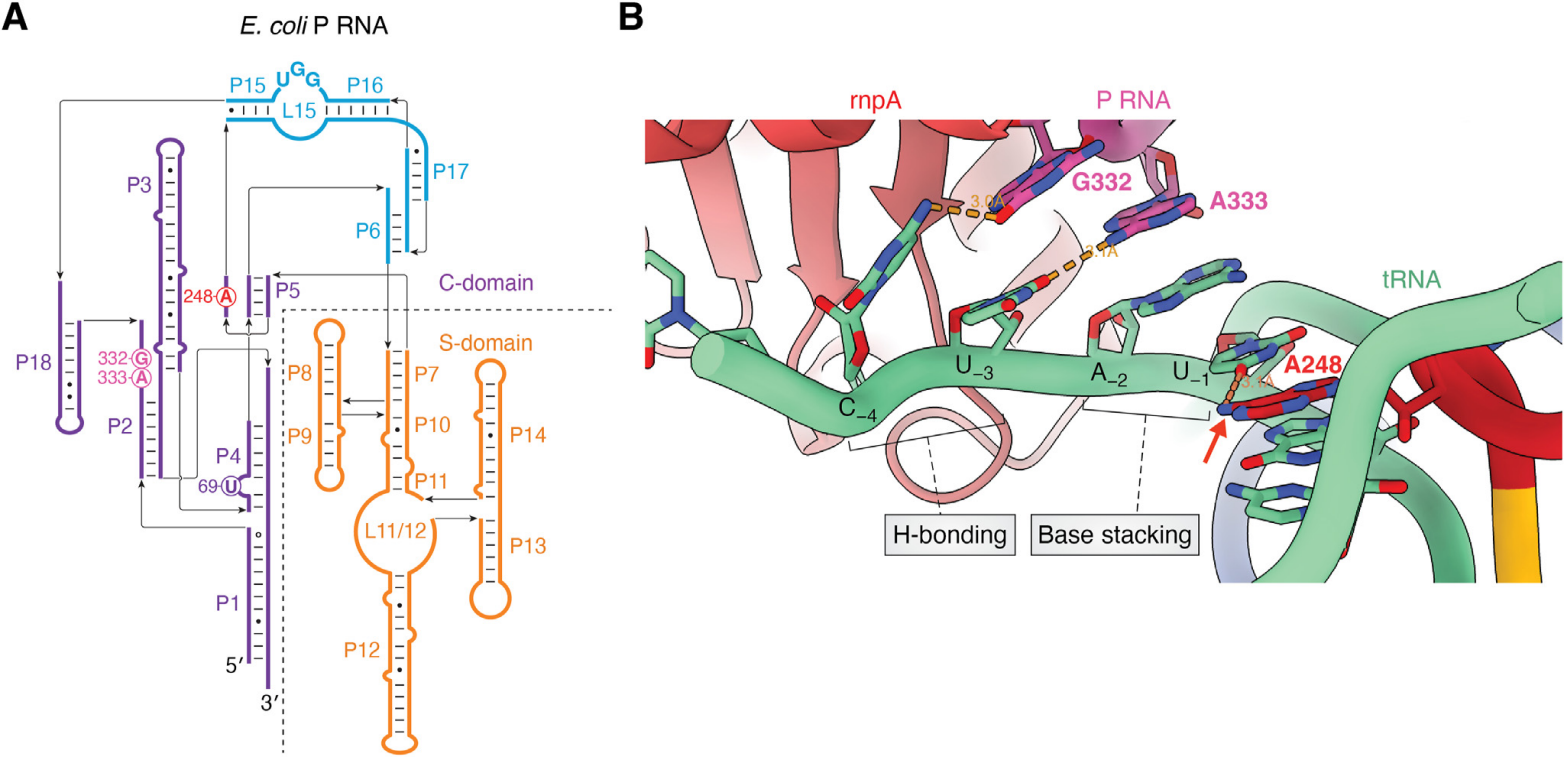
**The structural and mechanistic basis for alternative substrate recognition by bacterial Type A RNase P.***A*, secondary structure diagram of *E. coli* P RNA. *B*, close-up view of active site interactions and position of key P RNA residues. 5′ leader and tRNA (*light green*) and active site residues–A248 (*red*), G332, and A333 (*magenta*) are indicated.

The 4.1 Å crystal structure of the *T. maritima* RNase P bound to product tRNA was a milestone in RNA biology yet raised important questions about how the cleavage-site phosphodiester and proximal leader sequences are positioned in the active site (40). Recent cryo-EM structures of *E. coli* RNase P bound to an optimal pre-tRNA and a pre-tRNA with an extended acceptor stem showed how the active site accommodates sequence variation at the cleavage site for an optimal pre-tRNA (Fig. 5) and provided insight into conformational changes that occur during binding (41). The two otherwise identical pre-tRNA^met^ substrates interrogated in these structures were selected from HTS-Kin results. They contained either optimal A(-2)U(-1) at the cleavage site or G(-2)G(-1) that reacts with a 10-fold slower *k*_cat_/*K*_m_ due to base-pairing between these nucleotides and the 3′RCC which extends the acceptor stem in the free pre-tRNA. In both structures, the density map shows that when pre-tRNA is bound N(-1) pairs with the Hoogsteen face of A248 of P RNA (Fig. 5*B*) consistent with biochemical data (43, 44), and the 3′ RCCA forms well-established pairing interactions with the P15/16 internal bulge (93). Interestingly, different sequences at N(-1) and N(-2) positions are accommodated primarily by base-stacking that involves A333 and G332 in J18/2 of the P RNA subunit (*E. coli* numbering). The density map suggests that Hoogsteen edges of these residues would contact the nucleobases of N(-3) and N(-4) in the 5′ leader of pre-tRNA. Although the molecular details are unclear, these interactions could help define the basis for the nucleobase specificity at N(-4) revealed by HTS-Kin and mutagenesis studies.

**Figure 6:**
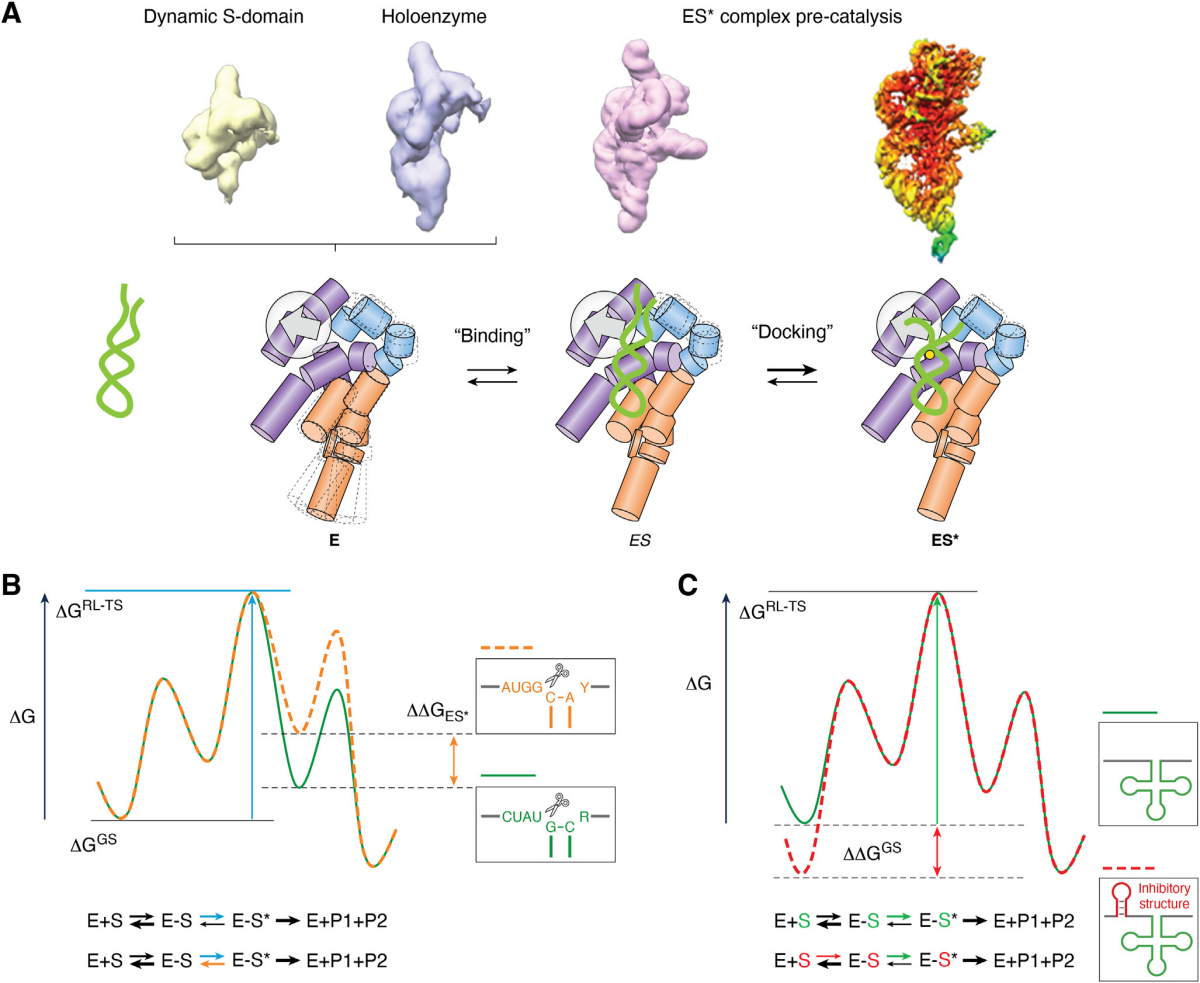
**Kinetic mechanism of RNase P multiple substrate recognition.***A*, evidence for flexible S-domain in the free *E. coli* RNase P holoenzyme and highly ordered ES∗ complex provides additional support for an induced-fit binding mechanism and insight into specific conformational changes. As described in the text, the available data support a two-step mechanism involving an encounter complex (ES) that undergoes one or more conformational changes before stabilization of the catalytically active ES∗ complex. *B*, simple kinetic scheme for cleavage of an optimal (*green*) and non-optimal (*orange*) pre-tRNA substrate by *E. coli* RNase P illustrates how an induced-fit mechanism can compensate for or mask differences in the strength of interactions with optimal specificity determinants. In this specific scenario, based on the cleavage of an optimal pre-tRNA^met^, the rate constant for undocking from the ES∗ complex is slow relative to catalysis. However, differences in transition state free energy (ΔG^RL-TS^) affected by the conformational change will contribute to specificity by affecting *k*_cat_/*K*_m_. *C*, scheme illustrating inhibitory structural determinants that affect the free substrate ensemble to influence the formation of ES. The difference in lowering the ground state free energy of the free ensemble (ΔΔG^GS^) contributes to the observed *k*_cat_/*K*_m_ and, therefore, affects specificity.

The cryo-EM data for the *E. coli* RNase P holoenzyme and ES∗ complex provided further insight into the conformational changes that may be coupled for accommodation and unpairing of 5′ leader-RCCA interactions. The structures suggest that binding a folded pre-tRNA to the T-loop induces motion that would position pre-tRNA into the active site (C-domain) and the L15 internal loop, capturing the 3′ RCCA. Notably, several bases surrounding the active site, such as A248, G332, and A333, have well-defined features in the holoenzyme structure, indicating a stable pocket pre-organized before 5′ leader sequence binding. The motion involving the S-domain could allow A248 and the stacking interactions with A333 to trap the separated single-base-pairs of the extended acceptor stem, allowing the termini of pre-tRNA to occupy the 5′-leader binding pocket and the 3′ RCCA at the L15 internal loop. This model is consistent with evidence for intrinsic conformational flexibility in P RNA involving the reorganization of the S- and C-domains inferred from X-ray scattering, SHAPE, and molecular modeling data (94). Time-resolved fluorescence resonance energy transfer between labeled rnpA protein and pre-tRNA 5′ leader further defined an unusually extended and relatively static RNA conformation for nucleotides proximal to the cleavage site (95). As such, the extended RNA conformation is consistent with RNase P acting as a wedge to separate the 5′ leader from the 3′ terminus of the pre-tRNA (95). Moreover, inter-strand crosslinks in the acceptor stem and near the cleavage site restrict 3′RCCA unpairing and reduce processing by *E. coli* RNase P (96). Thus, cooperation between RNA and protein subunits helps to offset the absence of recognition elements in the pre-tRNA. A consistent explanation is that free RNase P exists as a dynamic ensemble with motion between the domains emerging from the intrinsic dynamics of the S-domain. Pre-tRNA binding redistributes the ensemble of RNase P conformations and intermolecular and intramolecular interactions involving both subunits and including the binding of active site metal ions, then drives the formation of ES∗ (Fig. 6*A*).

In the ES∗ complex, pre-tRNA is bound in a distorted conformation, which is necessary for the extended acceptor stem to be unwound and gain access to the active site. The conformational changes needed for ES∗ formation thus provide additional free energy barriers that differ subtly depending on the respective pre-tRNA sequence. These conformational changes could contribute to the proposed induced fit mechanism. When catalysis is fast relative to the undocking of ES∗ back to ES, differences in the rate constant for undocking can have little effect on *k*_cat_/*K*_m_ for alternative substrates that differ with respect to active site interactions (Fig. 6*B*). In this case, the conformational change acts to suppress the effect of substrate variation on cleavage rate. However, *k*_cat_/*K*_m_ and, in turn, specificity between substrates is altered if the free energy of the rate-limiting transition state is affected, for example, due to a higher barrier for unwinding base pairing in an extended acceptor stem. For some cognate pre-tRNA, inhibitory structures can form in the free conformational ensemble that limits the formation of ES (Fig. 6*C*). An example is base pairing between upstream 5′ leader sequences and nucleotides near the cleavage site, such as those observed in multiple pre-tRNAs, including polycistronic transcripts (25). For such substrates, ground state effects or rate-limiting formation of ES can alter *k*_cat_/*K*_m_ and contribute to specificity for otherwise identical tRNAs.

## Emerging areas for exploration

RNase P is among a small subset of genes that are indispensable for bacterial survival (97). While the catalytic core of the P RNA subunit is conserved, the structure and composition of RNase P are different in the three domains of life (3, 40, 47, 86, 87, 88, 98, 99). Bacterial RNase P has features that are essentially identical as well as distinct from the human enzyme (87). RNase P has been recognized as a promising antibiotic target and continued effort is needed to realize its therapeutic potential (100). Avenues for bacterial RNase P inhibition that are advanced by a deeper understanding of molecular recognition include competitive inhibition, trapping inactive conformations, blocking substrate-induced conformational changes, and Mg^2+^ displacement (100, 101). Moreover, the detailed understanding of reaction kinetics and specificity provides a foundation for assay development and determining mechanisms of action. Aminoglycosides were recognized early on as inhibitors of bacterial RNase P (102), and can act by several mechanisms, including metal ion displacement (103) as well as binding to pre-tRNA and causing inhibition by substrate masking (104, 105, 106). Two studies reported the discovery of small-molecule inhibitors of bacterial RNase P by high-throughput screening (107, 108). A fluorescence polarization/anisotropy assay using full-length pre-tRNA was developed that relies on at least a two-fold change in signal when a 5-nt leader sequence is cleaved from an 82-nt pre-tRNA (108). Synthetic substrates derived from minihelices or bipartite pre-tRNAs severed at the anticodon loop have also been employed with end-attached fluorophore/quencher pairs. However, promising RNase P targeting drugs were recently identified as protein aggregators that do not inhibit RNase P with specificity, which calls into question their mode of action *in vivo* (109).

Despite all we know about bacterial RNase P specificity for pre-tRNA, a complete description of all its alternative substrates is lacking (65, 110). Foundational studies by the Altman lab established that RNase P can process alternative non-pre-tRNA substrates (66). The recent transcriptomic analysis by the Kushner laboratory, described above, represents a milestone in understanding the broad specificity and roles of RNase P. Now, there is a critical need to unravel direct effects and identify novel RNase P processing sites. In this regard, it is notable that ssRNA cleavage documented by Hartmann and colleagues suggests there could indeed be alternative substrates that may not resemble pre-tRNA (111). Similarly, *in vitro* selected substrates developed by the Pan laboratory showed that, at least in principle, there can be substrates that interact with RNase P in ways different from pre-tRNA (112). Using yeast RNase P, Engelke and colleagues showed that various mixed-sequence RNAs have multiple preferential cleavage sites that do not correspond to identifiable consensus structures or sequences for RNase P (113). Yeast nuclear but not bacterial RNase P is inhibited by homopolymer and other non-pre-tRNA alternative substrates, a finding that indicates differences in their respective specificities. These results suggest that there may be alternative binding modes yet to be uncovered for RNase P and indicate that models based on pre-tRNA data alone may miss alternative substrates. Therefore, a more general model is required that incorporates information from transcriptomics and a deeper investigation of non-pre-tRNA substrates.

Compared to the bacterial enzyme, the structure and biological roles of eukaryotic RNase P are more complex, but the understanding of its specificity and kinetics is less well-developed. Compared to the relatively small number of tRNA genes in bacteria (30–100), the nuclear genomes of eukaryotes have a much larger suite (a mean of ∼400, although some species encode as many as 15,000) (114). Also, tRNAs are integrated into multiple gene expression pathways, not just translation, and levels of tRNAs and tRNA modifications are important for cell function. With respect to tRNA biosynthesis, in *B. subtilis* and *Saccharomyces cerevisiae,* the turnover of mature tRNA occurs with little specificity and on long timescales. At the same time, there is evidence of significant turnover of pre-tRNA (35, 115). Since alterations to tRNA pools and specific tRNAs can contribute to disease including neurological disorders and cancer (116), extending quantitative modeling of RNase P specificity and other RNA processing enzymes represents a key future challenge. The competition between processing and turnover of pre-tRNA may represent a mechanism of RNA surveillance (35, 117, 118, 119). In this scenario, the ability of RNase P to recognize tRNA structure results in rapid 5′ processing of correctly folded pre-tRNA and accumulation of misfolded species, increasing the probability of their turnover. These factors underscore the importance of continued quantitative mechanistic investigation of eukaryotic and archaeal RNase P specificity and alternative substrate kinetics, especially since they can be interpreted in the context of available structural models.

Applications of high-throughput biochemical methods to endonucleases or RNA processing reactions like HTS-Kin further illustrate the importance of continued development and application. Lietard *et al*. probed the specificity of *E. coli* RNase HII using chemical photolithography to synthesize extensive combinatorial libraries of fluorescently labeled DNA/RNA chimeric sequences that self-anneal to form hairpin structures as substrates (120). The ability to identify sites of modification or cleavage significantly enhances the amount of information gained. For example, sequence and structure specificity was defined by combining a conventional aniline cleavage assay with high-throughput sequencing to study sequence-specific depurination of oligonucleotides caused by saporin (121). In a milestone application of deep mutational scanning of RNA substrate specificity, Fang and Bartel determined Dicer cleavage efficiencies of >50,000 variants of three human pri-miRNAs. Using a barcode strategy, they could reconstruct the sequence of precursors and cleavage sites by sequencing the 5′ cleavage product (122). These and similar applications have proven highly valuable for defining RBP and RNase specificity, and extending them to map specificity landscapes *in vivo* represents an exciting new frontier.

## Conflict of interest

The authors declare that they have no conflicts of interest with the contents of this article.
